# An amphipathic α-helix directs palmitoylation of the large intracellular loop of the sodium/calcium exchanger

**DOI:** 10.1074/jbc.M116.773945

**Published:** 2017-04-21

**Authors:** Fiona Plain, Samitha Dilini Congreve, Rachel Sue Zhen Yee, Jennifer Kennedy, Jacqueline Howie, Chien-Wen Kuo, Niall J. Fraser, William Fuller

**Affiliations:** From the Division of Molecular and Clinical Medicine, School of Medicine, Ninewells Hospital, University of Dundee, Dundee DD1 9SY, Scotland, United Kingdom

**Keywords:** acyltransferase, calcium transport, protein acylation, protein palmitoylation, sodium transport, sodium-calcium exchange

## Abstract

The electrogenic sodium/calcium exchanger (NCX) mediates bidirectional calcium transport controlled by the transmembrane sodium gradient. NCX inactivation occurs in the absence of phosphatidylinositol 4,5-bisphosphate and is facilitated by palmitoylation of a single cysteine at position 739 within the large intracellular loop of NCX. The aim of this investigation was to identify the structural determinants of NCX1 palmitoylation. Full-length NCX1 (FL-NCX1) and a YFP fusion protein of the NCX1 large intracellular loop (YFP-NCX1) were expressed in HEK cells. Single amino acid changes around Cys-739 in FL-NCX1 and deletions on the N-terminal side of Cys-739 in YFP-NCX1 did not affect NCX1 palmitoylation, with the exception of the rare human polymorphism S738F, which enhanced FL-NCX1 palmitoylation, and D741A, which modestly reduced it. In contrast, deletion of a 21-amino acid segment enriched in aromatic amino acids on the C-terminal side of Cys-739 abolished YFP-NCX1 palmitoylation. We hypothesized that this segment forms an amphipathic α-helix whose properties facilitate Cys-739 palmitoylation. Introduction of negatively charged amino acids to the hydrophobic face or of helix-breaking prolines impaired palmitoylation of both YFP-NCX1 and FL-NCX1. Alanine mutations on the hydrophilic face of the helix significantly reduced FL-NCX1 palmitoylation. Of note, when the helix-containing segment was introduced adjacent to cysteines that are not normally palmitoylated, they became palmitoylation sites. In conclusion, we have identified an amphipathic α-helix in the NCX1 large intracellular loop that controls NCX1 palmitoylation. NCX1 palmitoylation is governed by a distal secondary structure element rather than by local primary sequence.

## Introduction

Transmembrane calcium movement by electrogenic sodium/calcium exchangers (NCX)^2^ is a vital signaling event in multiple tissues. By controlling calcium dynamics in both smooth and striated muscle, for example, NCX is an important regulator of the contractility of skeletal, cardiac, and vascular smooth muscle. NCX can mediate either calcium extrusion (forward mode) or calcium influx (reverse mode) depending on the prevailing sodium and calcium gradients and the membrane potential. Three NCX isoforms exist in mammalian genomes. NCX1 is widely expressed and has multiple splice variants (NCX1.1 is the principal variant in cardiac muscle (1)). NCX2 and NCX3 are expressed in brain and skeletal muscle (2).

The NCX1 transmembrane architecture consists of two inverted repeats, each composed of five membrane-spanning helices (3, 4). The transmembrane domains are sufficient for exchanger activity (5), and regulation is mediated by a large (∼550 residues) intracellular loop between transmembrane domains 5 and 6. Calcium binding to two calcium-binding domains (CBDs) (CBD1, residues 370–501 in the mature exchanger, and CBD2, residues 501–599) facilitates calcium activation of NCX1 (6, 7). Sodium inactivates NCX1 (8), and this requires a region at the N-terminal end of the intracellular loop named the exchanger inhibitory peptide (XIP domain, residues 219–238 in the mature exchanger (9)). XIP itself inhibits NCX1 and is proposed to interact directly with a region encompassed by residues 562–679 (including part of CBD2, at the C-terminal end of the large intracellular loop) to achieve this (10).

Palmitoylation is the reversible attachment of a fatty acid (most commonly palmitate) to a cysteine sulfhydryl via a thioester bond. By recruiting the palmitoylated cysteine to the inner face of the phospholipid membrane, palmitoylation has the potential to impose significant structural and therefore functional changes on proteins (11). Many different types of proteins are palmitoylated; palmitoylation can regulate membrane association, cellular distribution, trafficking, and turnover, as well as enzymatic activity, phospholipid interactions, and channel/transporter activity (11–13). NCX1 is palmitoylated at a single cysteine at position 739 of its large intracellular loop (14). Exchangers that cannot be palmitoylated do not inactivate normally, leading to substantial activity in conditions when wild-type exchangers are inactive (14, 15). The NCX1 palmitoylated cysteine is conserved among all NCX isoforms in vertebrates.

Limited structural information is available for the NCX1 regulatory intracellular loop. X-ray and NMR structures of CBD1 and CBD2 both in isolation and together provide insight into calcium regulation of NCX1 (7, 16–18), but the region containing the palmitoylation site is essentially uncharacterized structurally. The section of the loop immediately C-terminal to the palmitoylation site was originally proposed to form transmembrane helix 6 of NCX1, but its localization was revised to cytosolic based on cysteine accessibility assays (19). A crystal structure of a bacterial homologue of NCX subsequently confirmed this and identified amino acid 761 as the start of transmembrane domain 6 of human NCX1 (4).

Like full-length NCX1, a YFP fusion protein of the NCX1 large intracellular loop is palmitoylated at Cys-739, indicating palmitoylation occurs independent of the presence of the NCX1 transmembrane domains (14) and that the palmitoylation “signal” for Cys-739 resides within this loop. Although there are multiple cysteines in the NCX1 intracellular loop, only Cys-739 is palmitoylated. The aim of this investigation was to identify the sequence determinants in the NCX1 intracellular loop that direct palmitoylation of Cys-739. We report that the section of this loop previously annotated as transmembrane domain 6 is required for NCX1 palmitoylation, and we suggest that the hydrophilic face of this largely hydrophobic α-helix is recognized by the cellular palmitoylation machinery.

## Results

### Amino acid substitutions close to the NCX1 palmitoylation site are largely without effect on NCX1 palmitoylation

**Figure 1.**
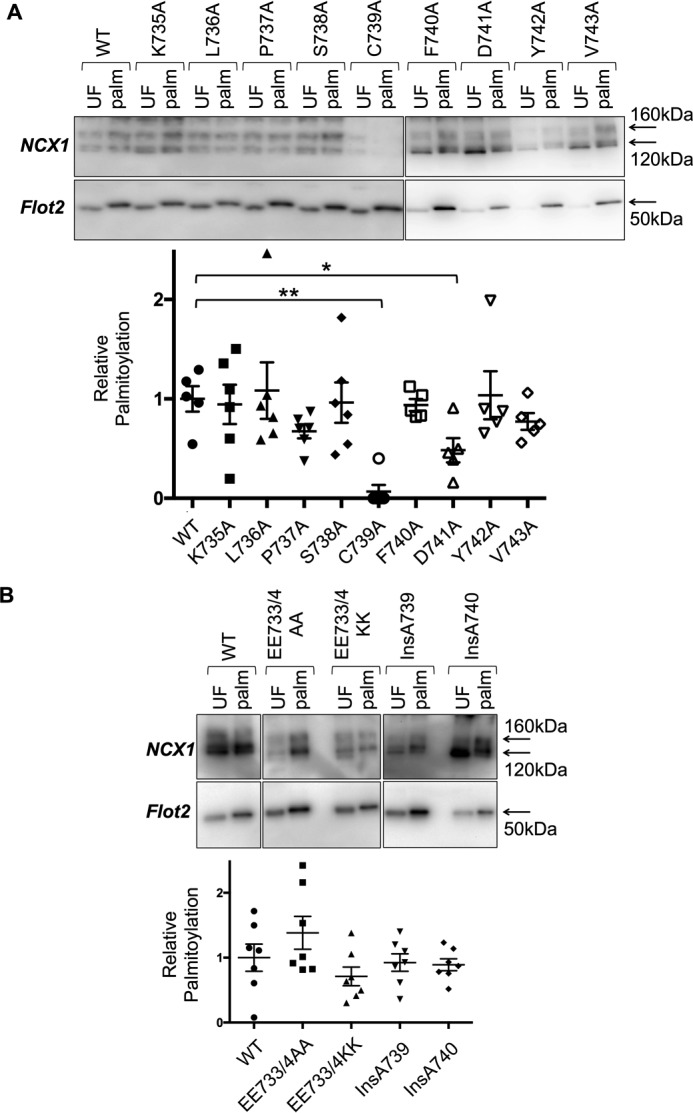
**Palmitoylation of NCX1 is largely independent of primary sequence close to the palmitoylation site.** Palmitoylated proteins were purified by resin-assisted capture and immunoblotted alongside their respective unfractionated cells lysates. In all experiments palmitoylation is expressed relative to expression. *A,* single amino acid substitutions with alanine do not influence NCX1 palmitoylation with the exception of D741A, which modestly reduces palmitoylation. *B,* charge substitutions and insertions before or after Cys-739 are without impact on NCX1 palmitoylation. *UF,* unfractionated cell lysate; *Palm,* palmitoylated proteins. *, *p* < 0.05 *versus* WT; **, *p* < 0.01 *versus* WT, *n* = 5–6.

Post-translational modifications such as phosphorylation is usually dictated by protein kinases recognizing primary sequence features local to the phosphorylation site. Palmitoylation prediction algorithms based on the primary sequence will only be valuable if the same recognition principle applies to acyltransferases. We therefore investigated whether changes to the primary sequence around the NCX1 palmitoylation site impaired the ability of the NCX1 acyltransferase to recognize and palmitoylate Cys-739. We first evaluated the effect of changing the amino acids surrounding Cys-739 to alanine on palmitoylation of full-length NCX1. Mutants K735A, L736A, P737A, S738A, F740A, D741A, Y742A, and V743A of NCX1 were assessed alongside wild-type and C739A (unpalmitoylatable) NCX1. With the exception of D741A, which displayed moderately reduced palmitoylation compared with wild type, none of the mutations affected NCX1 palmitoylation (Fig. 1*A*).

We also evaluated the impact of neutralizing (E733A/E734A) negative charges located N-terminal to Cys-739 or replacing them with positive ones (E733K/E734K) on NCX1 palmitoylation (Fig. 1*B*). In addition, we inserted an alanine either immediately before (InsA739) or immediately after (InsA740) the palmitoylated cysteine, changing the relative position of either all “upstream” or all “downstream” residues (Fig. 1*B*). Once again, all substitutions and insertions had no effect on the palmitoylation of Cys-739, strongly implying that in contrast to protein kinases the primary sequence surrounding Cys-739 has negligible impact on its palmitoylation.

### Single mutation adjacent to the NCX1 palmitoylation site increases NCX1 palmitoylation

**Figure 2.**
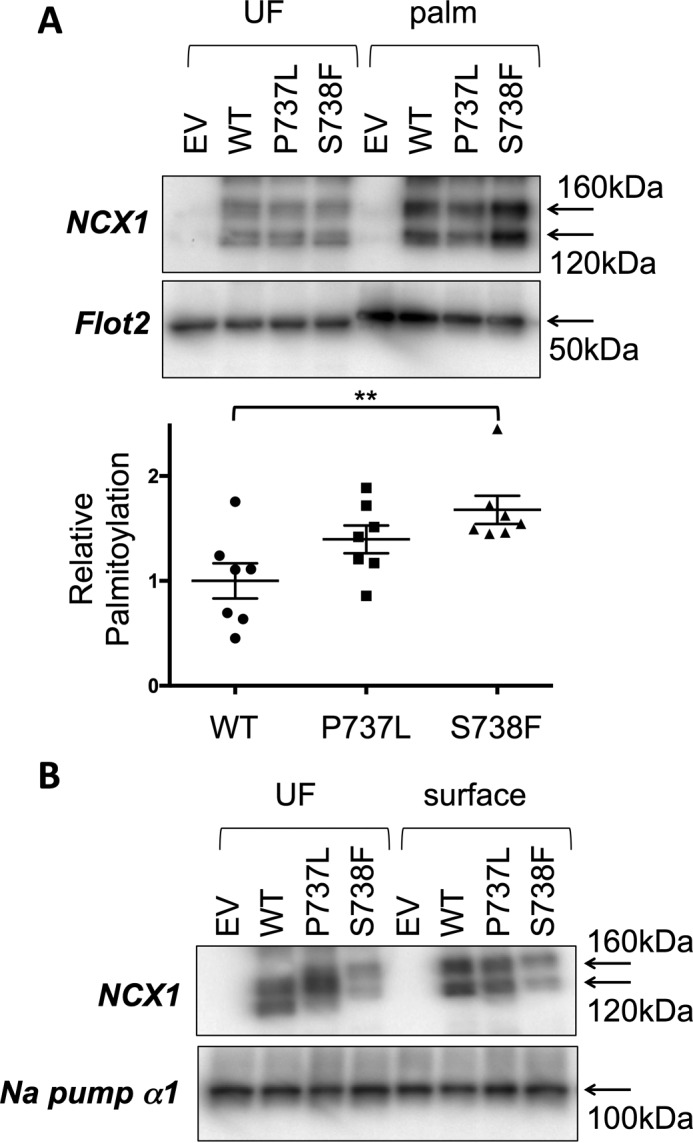
**Palmitoylation and trafficking of human polymorphisms of NCX1.** *A,* substitution of Ser-738 with a bulky phenylalanine residue increases the fraction of NCX1 palmitoylated in HEK cells, but P737L is without effect. *B,* polymorphisms P737L and S738F are without impact on the delivery of NCX1 to the cell surface. *EV,* empty vector transfected cells; *UF,* unfractionated cell lysate; *palm,* palmitoylated proteins **, *p* < 0.01 *versus* WT, *n* = 7.

We next investigated the impact of two validated but rare single nucleotide polymorphisms (SNPs) of human NCX1 on its palmitoylation. SNPs rs371485750 and rs373510583 generate the missense mutations P737L and S738F, respectively, albeit with very low minor allele frequencies. Substitution of Ser-738 with a bulky phenylalanine residue increased the fraction of full-length NCX1 palmitoylated at Cys-739 in HEK cells compared with wild type, whereas replacement of Pro-737 with leucine was without effect (Fig. 2*A*). Neither mutation prevented the expression of NCX1 at the cell surface assessed using membrane-impermeable biotinylation reagents (Fig. 2*B*).

### Amphipathic α-helix distal and C-terminal to Cys-739 is required for NCX1 palmitoylation

**Figure 3.**
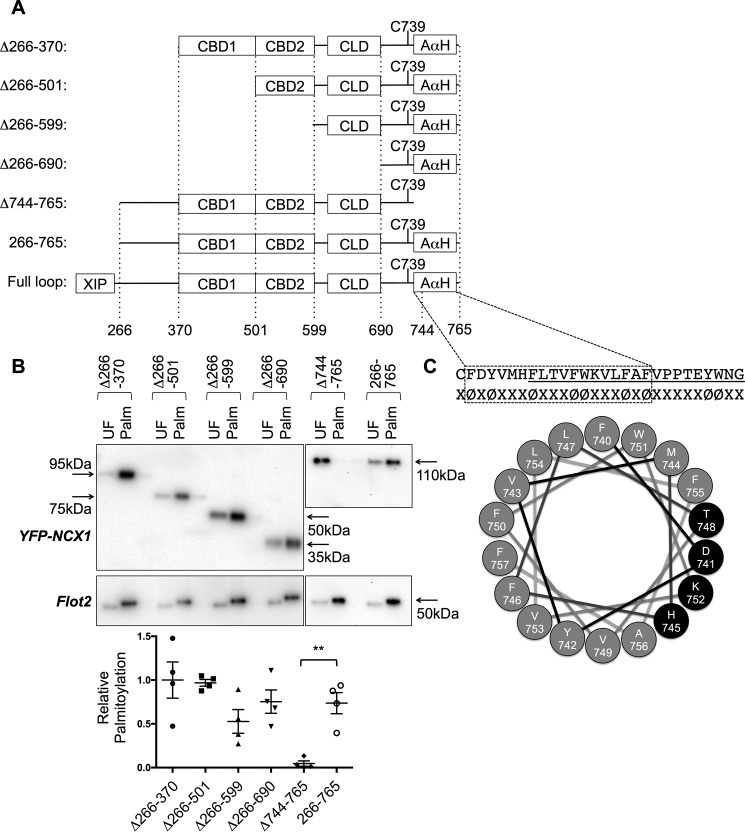
**Palmitoylation of YFP-NCX1 requires an amphipathic α-helix on the C-terminal side of the palmitoylation site.** *A,* schematic of the NCX1 intracellular loop, indicating the positions of the calcium-binding domains (*CBD*), catenin-like domain (*CLD*), and amphipathic α-helix (*A*α*H*). The positions of truncations are indicated *below* the schematic. *B,* impact of truncations on the palmitoylation of YFP-NCX1. Only removal of amino acids 744–765 causes palmitoylation of Cys-739 to be abrogated. *C,* sequence of NCX1 residues 739–765, highlighting the abundant aromatic amino acids (φ). The *underlined region*, 744–765, is required for palmitoylation of NCX1 in *B*. The *boxed region* is predicted to form an α-helix by JPred4. A projection of this α-helix is shown, with the polar face highlighted in *black. UF,* unfractionated cell lysate; *Palm,* palmitoylated proteins. **, *p* < 0.01 *versus* 266–765 (WT), *n* = 5.

Having established that the local primary sequence has a negligible effect on NCX1 palmitoylation, we sought to identify those regions within the NCX1 intracellular loop required for its palmitoylation by truncating the loop from both the N and C termini (Fig. 3*A*). A YFP fusion protein with the NCX1 intracellular loop (amino acids 266–765, from the end of the XIP domain to TM6) is palmitoylated in HEK cells (Fig. 3*B*). N-terminal truncations successively removed the region between XIP and CBD1 (residues 266–370), CBD1 (residues 370–501), CBD2 (residues 501–599), and the proposed catenin-like domain (17) adjacent to CBD2 (residues 599–690). All were without effect on the palmitoylation of YFP-NCX1 (Fig. 3*B*). In contrast, removal of 21 amino acids on the C-terminal side of the NCX1 palmitoylation site (residues 744–765) completely abolished NCX1 palmitoylation in HEK cells (Fig. 3*B*).

**Figure 4.**
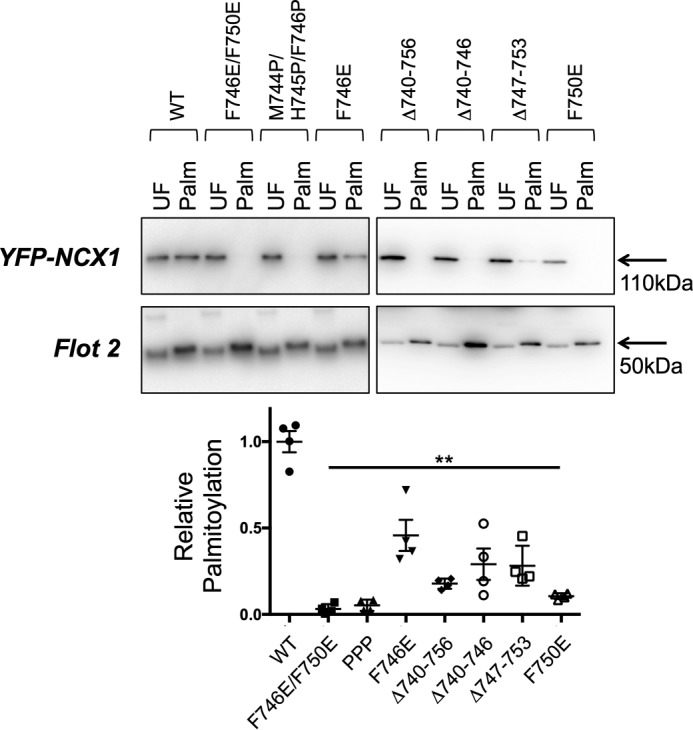
**Palmitoylation of YFP-NCX1 is abolished by mutations in the C-terminal α-helix.** Introduction of a single negative charge at position 746 (F746E) reduces but does not eliminate palmitoylation of YFP-NCX1, whereas palmitoylation is completely abolished either by introducing a negative charge in position 750 (F750E), two negative charges (F746E/F750E), or the helix-breaking insertion of three prolines (M744P/H745P/F746P). Deletion of the entire α-helix (Δ740–756), the first two turns of the helix (Δ740–746), or the second two turns of the helix (Δ747–753) also prevents or reduces YFP-NCX1 palmitoylation. *UF,* unfractionated cell lysate; *Palm,* palmitoylated proteins. **, all *p* < 0.01 *versus* WT, *n* = 4.

Residues 745–765 of NCX1 include a region initially annotated as a transmembrane helix, whose cytosolic location was later established by cysteine accessibility assays (19). The secondary structure prediction algorithm Jpred4 (20) suggests with high confidence that residues 740–757 of NCX1 form an α-helix (Fig. 3*C*). A helical wheel projection (Fig. 3*C*) indicates that this helix is expected to have a small hydrophilic face (residues *D741, H745, T748,* and *K752 highlighted in black*), with the remainder of the helix hydrophobic in character and rich in aromatic amino acids, particularly on one face (*F746, F750,* and *F757*). We hypothesized that the amphipathic nature of this helix but (given the lack of impact of mutant InsA740 on Cys-739 palmitoylation) not its location was critical for palmitoylation of NCX1. To explore this possibility, a series of mutagenesis experiments were performed. Mutation of aromatic amino acids within the proposed helix to alanine either individually or in combination did not alter palmitoylation of YFP-NCX1 (not shown: Y742A, F746A, F750A, W751A, and F755A). Next, we assessed the impact of introducing helix-breaking prolines and membrane-repelling glutamates to this helix on the palmitoylation of YFP-NCX1 (residues 266–765). Introduction of a single negative charge at position Phe-746 (F746E) reduced but did not abolish palmitoylation of YFP-NCX1, whereas either introducing a single negative charge at position Phe-750 (F750E) or introducing three helix-breaking prolines (M744P/H745P/F746P) both abolished YFP-NCX1 palmitoylation (Fig. 4). Deletion of either the whole proposed helix (Δ740–756), the first two turns (Δ740–746), or the second two turns (Δ747–753) also abolished palmitoylation of YFP-NCX1 (Fig. 4), suggesting the whole α-helix is the minimum region required to direct YFP-NCX1 palmitoylation.

### Amphipathic α-helix on the C-terminal side of the NCX1 palmitoylation site is required for palmitoylation but not trafficking of full-length NCX1

**Figure 5.**
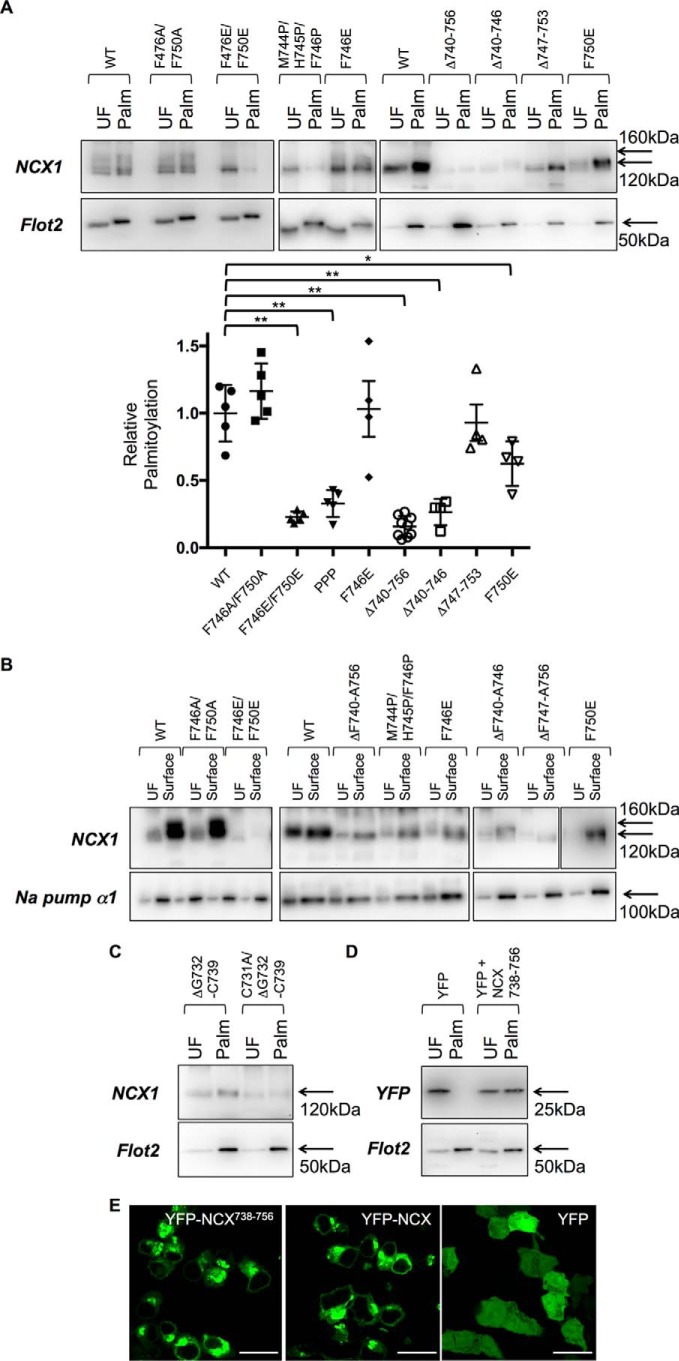
**Point mutations and palmitoylation of full-length NCX1.** *A,* point mutations F746E/F750E, Δ740–756, Δ740–746, and M744P/H745P/F746P prevent palmitoylation of full-length NCX1, whereas F750E reduces full-length NCX1 palmitoylation, and Δ747–753 and F746E are without effect. *B,* of the mutations assessed in *A*, only F746E/F750E impedes progression of full-length NCX1 through the secretory pathway. *C,* NCX1 is palmitoylated at Cys-731 when the region usually on the C-terminal side of Cys-739 is instead positioned adjacent to Cys-731. *D,* C-terminal fusion of NCX1 residues 738–756 is sufficient to direct palmitoylation of YFP. *E,* C-terminal fusion of NCX1 residues 738–756 anchors YFP to intracellular membranes in a manner indistinguishable from YFP-NCX1. *Scale bar,* 20 μm. *, *p* < 0.05 *versus* WT; **, *p* < 0.01 *versus* WT, *n* = 5.

We next investigated the impact of the mutations characterized in Fig. 4 on palmitoylation and trafficking of full-length NCX1 (FL-NCX1). Although palmitoylation is not required for passage of NCX1 through the secretory pathway (14), mutations disrupting the endoplasmic reticulum exit through misfolding of FL-NCX1 would reduce palmitoylation, as NCX1 is palmitoylated in the Golgi (14). Mutation F746E/F750E abolished palmitoylation of NCX1 (Fig. 5*A*), but it also prevented its delivery to the cell surface (Fig. 5*B*), suggesting that this mutant was misfolded. F746E and F746A/F750A were palmitoylated and trafficked normally, whereas mutant F750E reduced palmitoylation but not trafficking of FL-NCX1. No alanine mutations of aromatic amino acids within the proposed helix impacted palmitoylation of FL-NCX1 (not shown: F746A, F750A, W751A, and F755A). Deletion of the predicted amphipathic α-helix (Δ740–756) or breaking this α-helix (M744P/H745P/F746P) largely abolished palmitoylation but not cell surface delivery of FL-NCX1. Palmitoylation of FL-NCX1 was sustained in the absence of the second two turns of the proposed helix (Δ746–753, in contrast to YFP-NCX1) but abolished in the absence of the first two (Δ740–746).

### Amphipathic α-helix is capable of directing palmitoylation of cysteines that are not normally palmitoylated

We investigated the ability of the amphipathic α-helix required for palmitoylation of Cys-739 to “confer” palmitoylation on another cysteine within NCX1 that is not usually palmitoylated. By removing amino acids 732–739 from NCX1 (including the normal palmitoylation site) the putative helical recognition element was positioned immediately adjacent to Cys-731 (which is not usually palmitoylated). Fig. 5*C* indicates that the Δ732–739 NCX1 mutant was palmitoylated at Cys-731, despite the presence of seven consecutive negatively charged residues (^724^EDDDDDE^730^) on the immediate N-terminal side of Cys-731.

In an additional “gain-of-function” experiment, we added NCX1 residues 738–756 (including both palmitoylated cysteine and amphipathic α-helix) to the C terminus of YFP. As a result, YFP was palmitoylated (Fig. 5*D*) and tethered to intracellular membranes in a manner indistinguishable from YFP-NCX1 (Fig. 5*E*).

### Hydrophilic face of the amphipathic α-helix is required for palmitoylation of NCX1 Cys-731

**Figure 6.**
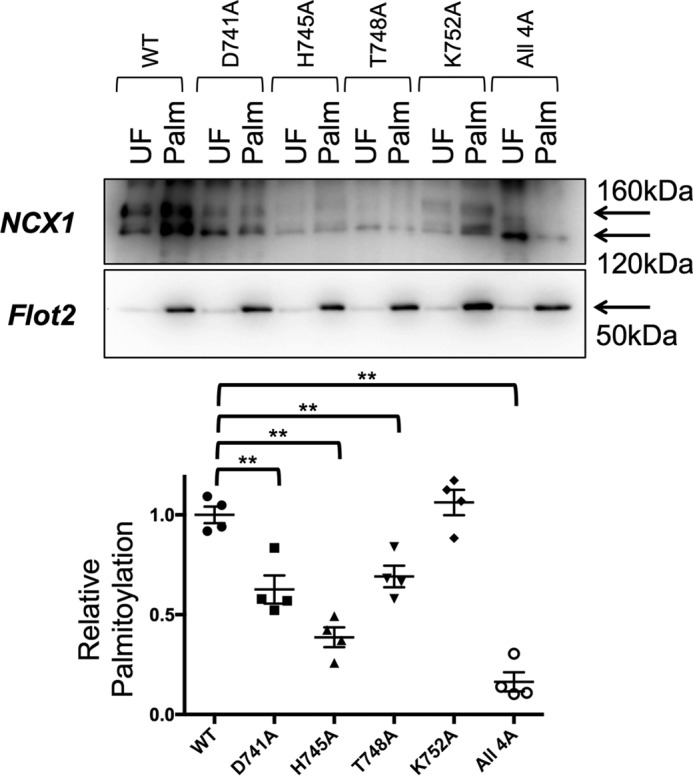
**Alanine mutations on the hydrophilic face of the amphipathic α-helix impair NCX1 palmitoylation.** Substitution of Asp-741, His-745, and Thr-748 but not Lys-752 reduce palmitoylation of full-length NCX1. Substitution of all four residues on the hydrophilic face with alanine (All4A: D741A/H745A/T748A/K752A) reduces NCX1 palmitoylation by ∼85%, *UF,* unfractionated cell lysate; *Palm,* palmitoylated proteins. **, *p* < 0.01 *versus* WT, *n* = 4.

We investigated the impact of changing the four residues on the hydrophilic face of the amphipathic α-helix to alanine. Substitution of Asp-741, His-745, Thr-748 but not Lys-752 with alanine reduced palmitoylation of FL-NCX1, with the largest reduction observed when His-745 was mutated. The most significant impact on FL-NCX1 palmitoylation was achieved by the substitution of all four residues on the hydrophilic face with alanine (D741A/H745A/T748A/K752A, Fig. 6); NCX1 palmitoylation was reduced by ∼85%, similar to the effect of removing the amphipathic α-helix altogether.

## Discussion

Here we identify the region of the NCX1 intracellular loop that directs its palmitoylation, as well as determining the properties of this region required for both palmitoylation and trafficking of NCX1. Palmitoylation of NCX1 is determined by secondary rather than primary structure; residues 740–756 are required for NCX1 to be palmitoylated. Disruption of either the α-helical geometry, the introduction of more than one negatively charged amino acid to the hydrophobic face, or alanine mutagenesis of the hydrophilic face of this helix all drastically reduce or abolish NCX1 palmitoylation. Interestingly this region is 100% identical in both NCX2 and NCX3, but with cysteines at both N- and C-terminal ends of the helix (analogous to positions 739 and 757 in NCX1). These cysteines would appear on the same face of the α-helix, which we suggest may support dual palmitoylation of these NCX family members.

Surprisingly, NCX1 residues 740–756 are dispensable for the trafficking of full-length NCX1 to the cell surface, but when present this region must be largely hydrophobic (no more than one additional negatively charged amino acid can be accommodated) but not necessarily α-helical (helix-breaking prolines do not abolish trafficking).

On first sight, our data may appear to be consistent with a model in which weak membrane interaction of a cytosolic region of a protein orients a cysteine appropriately for transfer of palmitate from a palmitoylating acyltransferase enzyme. The lack of impact of mutant InsA740 on NCX1 palmitoylation suggests the position of Cys-739 relative to this helix is unimportant. Indeed, the palmitoylation of substrates by Golgi-localized Asp-His-His-Cys-containing palmitoyl acyltransferases (DHHC-PATs) has been argued to be largely stochastic, without specific enzyme/substrate pairs (21). However, the deletion of residues 740–756 of full-length NCX1 will also position Cys-739 very close to the membrane (transmembrane domain 6 starts at position 761 (4)) but notably does not support its palmitoylation. Even in the presence of this amphipathic helix, unpalmitoylatable C739A YFP-NCX1 is not found membrane-associated (14). There is evidently greater specificity to the palmitoylation reaction for NCX1 than a simple random event. Certain DHHC-PATs recognize substrates via the formation of stable protein-protein interactions in regions of the enzyme separated from the catalytic site (22–25). Indeed, in some cases regions distant from the palmitoylated cysteine have been found to control palmitoylation (26). We therefore suggest that the hydrophilic face of NCX1 residues 740–756 is responsible for interaction with the palmitoylating DHHC-PAT. Consistent with this, positioning this region adjacent to NCX1 Cys-731 supports palmitoylation of this site and is sufficient to direct palmitoylation of YFP. The ability of the amphipathic α-helix identified in this investigation to drive palmitoylation of cysteines that are not normally palmitoylated may therefore have biotechnology applications, for example in reversibly anchoring soluble proteins to the Golgi apparatus. Equivalent motifs likely exist directing palmitoylation of particular cysteines by DHHC-PATs in other cellular compartments.

We find minor differences between the palmitoylation of YFP-NCX1 and full-length NCX1 that imply the presence of transmembrane domains (and possibly proximity to the membrane) can also influence palmitoylation of a particular cysteine. Mutants F746E and F750E are essentially unpalmitoylated in the absence of transmembrane domains, but F746E does not influence palmitoylation of full-length NCX1, and F750E only modestly reduces it. Similarly, deletion of the second half of the amphipathic α-helix (Δ747–753) abolishes palmitoylation of YFP-NCX1, but it is without effect on full-length NCX1. The importance of the second half of this helix for NCX1 palmitoylation is emphasized by the reduced palmitoylation in the presence of mutation T748A on the hydrophilic face. Nevertheless, our data indicate that proximity to the membrane (the presence of transmembrane domains) “loosens” the required consensus for NCX1 palmitoylation as follows: the first two turns of the helix are essential, but the absence of the second two turns can be mitigated by transmembrane domains positioning Cys-739 close to the membrane.

Several palmitoylation prediction tools developed in recent years have attempted to define a palmitoylation “consensus sequence” (27–29). Three recent studies have suggested hydrophobic residues on the N-terminal side and basic residues on the C-terminal side of a particular cysteine will favor its palmitoylation (28, 30, 31). Clearly, this is not the case for NCX1; the N-terminal side of the palmitoylation site is enriched in negatively charged amino acids (9 of the first 20 residues on the N-terminal side are aspartate or glutamate), and the carboxyl side is hydrophobic. Indeed, the fact that when positioned adjacent to Cys-731, NCX1 residues 740–756 are capable of inducing palmitoylation of this site (despite the abundant negative charges on the N-terminal side) emphasizing the difficulty of using palmitoylation predictors based on primary sequence. Substrate recognition by DHHC-PATs is clearly fundamentally different from protein kinases; phosphorylation sites can be accurately predicted based on primary sequence (although certain kinases and phosphatases also rely on interactions outside the active site (32)). As palmitoyl proteomes and palmitoylation sites are catalogued and DHHC-PATs are better characterized, the sequence rules for identifying palmitoylation sites may improve. Low specificity “stochastic” palmitoylation sites may well prove amenable to identification simply by inspecting primary sequences, but palmitoylation events that rely on stable interactions between DHHC-PAT and substrate will likely not, and will have to be determined experimentally. Because no mutations on the N-terminal side of Cys-739 impaired NCX1 palmitoylation, we suggest that the enhanced palmitoylation of the naturally occurring NCX1 polymorphism S738F may represent an impairment of NCX1 depalmitoylation. Most likely the flanking of the palmitoylated cysteine with bulky phenylalanines (Phe is also in position 740) impairs the ability of the NCX1 thioesterase to access the thioester bond at Cys-739. Hence, our data also suggest an experimental strategy for gain-of-function experiments to specifically enhance the palmitoylation of proteins. The use of negatively charged amino acids as phosphomimetic mutants is widespread in understanding the role of phosphorylation of individual residues. To date, such an approach has not been available to investigate the biology of protein palmitoylation.

In conclusion, we have identified an amphipathic α-helix at the C-terminal end of the NCX1 intracellular loop that controls palmitoylation of NCX1. Palmitoylation of NCX1, and likely other proteins, is directed by secondary rather than primary structure.

## Experimental procedures

### Antibodies

Anti-NCX1 was from Swant; anti-flotillin 2 was from BD Biosciences; anti-GAPDH was from Sigma; and anti-GFP was from Abcam. The monoclonal antibody α6F raised against the sodium pump α1 subunit by Douglas M. Fambrough was obtained from the Developmental Studies Hybridoma Bank developed under the auspices of the NICHD, National Institutes of Health, and maintained by the Department of Biology, University of Iowa.

### Purification of palmitoylated proteins

Palmitoylated proteins were purified using thiopropyl-Sepharose in the presence of neutral hydroxylamine after alkylation of free thiols with methyl methanethiosulfonate, as described previously (22).

### Purification of cell-surface proteins

Representative fractions of cell-surface proteins were prepared by treating cells with 1 mg/ml sulfo-NHS-SS-biotin (Pierce) for 10 min at 37 °C to biotinylate integral surface membrane proteins with extracellular primary amines, which were subsequently purified using streptavidin-Sepharose (GE Healthcare).

### Plasmids, cell lines, and transfection

The plasmids used were based on canine NCX1.1 and are described elsewhere (14). Amino acid numbering is for the cardiac splice variant NCX1.1 with the signal peptide removed. FL-NCX1 typically migrated on SDS-PAGE as bands 120 and 160 kDa in size, although the presence of the 160-kDa species was not consistent. All transfections of plasmid DNA used Lipofectamine 2000 (Invitrogen) according to the manufacturer's instructions.

### Site-directed mutagenesis

Mutagenesis reactions were performed with the Quikchange II site-directed mutagenesis kits (Agilent). Mutagenesis primers were designed using the Agilent on-line primer design tool.

### Microscopy

Images were acquired from cells fixed in 4% paraformaldehyde 24 h after transfection using a Leica SP5 confocal microscope. Acquisition parameters were (excitation/emission, nm) YFP, 519/542.

### Quantitative and statistical analysis

Western blottings were acquired using a ChemiDoc XRS imaging system and images quantitated using the QuantityOne software package (Bio-Rad). Band intensity in the “palmitoylated” fraction was normalized to the corresponding unfractionated cell lysate. To account for day-to-day variations in palmitoylation stoichiometry, individual data points were normalized to the group mean for that particular experimental day. Identical results were obtained by normalizing individual data points to the palmitoylation of wild-type NCX1 on that particular experimental day. Quantitative data are presented as means ± S.E. The differences between experimental groups were analyzed by one-way analysis of variance, followed by post hoc *t* tests. Differences were considered statistically significant with *p* < 0.05.

## Author contributions

F. P. performed and analyzed experiments shown in Figs. 1, 2, and 4–6. S. D. C. performed and analyzed experiments shown in Figs. 4 and 5. R. S. Z. Y. performed and analyzed experiments shown in Figs. 1 and 3. J. H. performed and analyzed experiments show in Figs. 1–3. J. K. and C.-W. K. performed and analyzed experiments shown in Fig. 5. W. F. and N. J. F. conceived the idea for the project and wrote the paper. All authors reviewed the results and approved the final version of the manuscript.

## Footnotes

2 The abbreviations used are:

NCX: sodium/calcium exchanger
FL-NCX1: full-length NCX1
CBD: calcium-binding domain
PAT: palmitoyl acyltransferase
SNP: single nucleotide polymorphism.

## Note added in proof

There was some textual overlap between the version of this article that was published as a Paper in Press on April 21, 2017, and Reilly, L., Howie, J., Wypijewski, K., Ashford, M. L., Hilgemann, D. W., and Fuller, W. (2015) *FASEB J.* **29,** 4532–4543. The overlap has now been removed.
